# Low Density Lipoprotein-Containing Circulating Immune Complexes: Role in Atherosclerosis and Diagnostic Value

**DOI:** 10.1155/2014/205697

**Published:** 2014-06-18

**Authors:** Igor A. Sobenin, Jukka T. Salonen, Andrey V. Zhelankin, Alexandra A. Melnichenko, Jari Kaikkonen, Yuri V. Bobryshev, Alexander N. Orekhov

**Affiliations:** ^1^Russian Cardiology Research and Production Complex, Moscow, Russia; ^2^Institute of General Pathology and Pathophysiology, Moscow, Russia; ^3^Hjelt Institute, University of Helsinki, Helsinki, Finland; ^4^MAS-Metabolic Analytical Services Oy, Helsinki, Finland; ^5^The Research Centre of Applied and Preventive Cardiovascular Medicine, University of Turku, Turku, Finland; ^6^Faculty of Medicine, School of Medical Sciences, University of New South Wales, Kensington, Sydney, NSW 2052, Australia; ^7^School of Medicine, University of Western Sydney, Campbelltown, NSW, Australia; ^8^Institute for Atherosclerosis Research, Skolkovo Innovation Center, Moscow, Russia

## Abstract

It has been suggested that low density lipoprotein-containing circulating immune complexes (LDL-CIC) play a role in atherogenesis and are involved in the formation of early atherosclerotic lesion. These complexes, as well as anti-LDL autoantibodies, have been found in the blood and in the atherosclerotic lesions of patients with different cardiovascular diseases, as well as in the blood of animals with experimental atherosclerosis. It can be suggested that the presence of anti-LDL antibodies in the blood is a result of immune response induced by lipoprotein modification. LDL-CIC differs from native LDL in many aspects. It has much lower sialic acid content, smaller diameter, and higher density and is more electronegative than native LDL. Fraction of LDL-CICs is fundamental to the serum atherogenicity manifested at the cellular level. LDL-CIC, unlike native LDL, is able to induce intracellular accumulation of neutral lipids, especially esterified cholesterol, in cells cultured from uninvolved human aortic intima and in macrophage cultures. After removal of LDL-CIC, the CHD patient's sera lose their atherogenic properties. Titer of LDL-CIC in blood serum significantly correlates with progression of atherosclerosis in human *in vivo* and has the highest diagnostic value among other measured serum lipid parameters. Elevated CIC-cholesterol might well be a possible risk factor of coronary atherosclerosis.

## 1. Introduction

Widely spread clinical manifestations of atherosclerosis such as coronary heart disease (CHD), cerebrovascular stroke, renovascular hypertension, and violation of the lower limbs vascular permeability, are the result of formation of advanced atherosclerotic lesions in a vascular wall. A trigger mechanism for the development of atherosclerotic lesions is an intracellular lipid deposition and subsequent foam cell formation with excessive production of connective tissue matrix components and, possibly, cellular proliferation and inflammatory reactions [1, 2]. Atherosclerosis can be generally described as an excessive fibrofatty, proliferative, inflammatory response to damage of the artery wall, involving several cell types, such as smooth muscle cells, monocyte-derived macrophages, lymphocytes, and platelets [3]. During the last three decades, the autoimmune hypothesis of atherosclerosis was developed and the evidence for an important role for autoantibodies against modified low density lipoprotein (LDL) and LDL-containing circulating immune complexes (LDL-CIC) in atherogenesis has been accumulated. Immunological factors appear to contribute to the development of atherosclerosis as many other factors including alterations in plasma lipid and lipoprotein levels, platelet function, clotting factors, arterial smooth muscle cell metabolism, and blood pressure regulation. In a number of recent studies it has been suggested that the presence of LDL-CIC in the blood promotes the onset and development of atherosclerotic lesions in the vessel wall. It has been demonstrated that modified LDL and especially LDL-CIC act as the primary agents responsible for excessive cholesterol accumulation in vascular cells [4–9]. The atherogenic properties of LDL-containing immune complexes suggest them as a candidate marker for atherosclerosis.

## 2. LDL-CIC and Its Physicochemical Characteristics

Anti-LDL autoantibodies were first detected in the blood of patients with hyperlipidemia accompanied by myeloma or/and ischemic heart disease [10]. In 1965, Beaumont [11] described a situation in which hyperlipidemia, xanthomatosis, and atherosclerosis were apparently associated with anti-*β*-lipoprotein antibodies. The antibodies against lipoproteins or LDL-binding factors were found in the blood of patients suffering from various vascular diseases as well as in healthy subjects [12]. Bauer et al. established that immunoglobulins are the major LDL-binding proteins in human plasma [13]. The emergence of anti-LDL autoantibodies in the blood implies that lipoproteins can be regarded as autoantigens. The high immunogenicity and ability of homologous chemically modified LDL to generate antibodies have been demonstrated [14]. The discovery of autoantibodies in modified LDL in the blood of patients therefore seems natural. Autoantibodies against glycosylated LDL were detected in the blood of patients with diabetes mellitus [15]. Autoantibodies specific for malondialdehyde-modified LDL have been found in the blood of healthy subjects and patients with coronary artery disease, as well as in the blood of experimental animals [16]. Deposits of the immune complex components were found in vascular atherosclerotic lesions [17–19]. Autoantibodies of immunoglobulin G class against modified LDL were detected in the blood of patients with angiographically assessed coronary atherosclerosis [2, 12, 20–23]. In healthy subjects, the level of anti-LDL autoantibodies was considerably lower than that in atherosclerotic patients [2]. These autoantibodies exhibit a high affinity for desialylated LDL (neuraminidase-treated LDL) and for malondialdehyde-modified LDL. They have a lower affinity for native, oxidized, glycosylated, and acetylated LDL, as well as for LDL, which has undergone other chemical modifications. The higher affinity of autoantibodies for modified LDL compared with native LDL suggests that the antibodies are produced* in vivo* in response to the appearance of modified LDL in the blood [24, 25].

Antibodies against LDL modified with malondialdehyde (MDA) have been detected in the blood of animals with experimental atherosclerosis and in atherosclerotic lesions in humans [25–28]. Even though elevated levels of oxidized lipids, such as MDA and F_2_-isoprostanes, have been found in the blood of subjects with CHD [29, 30], there is some evidence that oxidized lipids do not accumulate in noticeable amounts in human LDL since high density lipoproteins seem to detoxify and/or transfer them from the circulation to the liver [31]. On the other hand, electronegative LDL [32], small/dense LDL [33], and desialylated LDL differing from native LDL by lowered sialic acid content [2, 34, 35] were found in the blood of patients with coronary atherosclerosis.

It can be suggested that the presence of anti-LDL antibodies in the blood is a result of immune response induced by lipoprotein modification. Tertov et al. [36] isolated circulating immune complexes from blood serum using polyethylene glycol 6000 and have found that LDL-CIC differs from native LDL in many aspects (Table 1). Specifically, it has low sialic acid content; that is, it is desialylated LDL. The neutral lipid and phospholipid contents of LDL-CIC are considerably lower than those in native LDL. Particles of LDL-CIC have a smaller diameter and higher density. The higher electrophoretic mobility shows that LDL-CIC is more electronegative than native LDL. Finally, LDL-CIC, unlike native LDL, is able to induce intracellular accumulation of neutral lipids, especially esterified cholesterol, in cells cultured from uninvolved human aortic intima. Thus, it was shown that LDL-CIC is quite similar to the multiple-modified (desialylated) LDL described earlier [37–39]. There was a strong correlation between the LDL content in circulating immune complexes and blood concentration of desialylated LDL but not of total LDL. This suggests that predominantly desialylated LDL forms complex with autoantibodies and proves that the affinity of circulating anti-LDL autoantibodiesis higher for desialylated LDL than for native LDL [36]. Moreover, anti-LDL autoantibodies bind much more effectively with LDL of patients having a high percentage of desialylated LDL than with LDL of healthy subjects having a low content of desialylated LDL [40]. Desialylated LDL has certain modifications that could stimulate the immune response: alterations in carbohydrate composition and in the tertiary structure of apo B, modification of lysine amino groups, and aggregation of lipoprotein particles [38, 39].

## 3. Atherogenicity of LDL-CIC

The investigation of proatherogenic role of LDL-CIC has started long ago, and it seems relevant to look into historical perspective to revive the interest in this topic. The first experimental data on the effect of LDL and anti-LDL autoantibodies on cell metabolism were reported by Beaumont's group as far as in 1979 [41]. It was found that incubating cultured fibroblasts with LDL, forming immune complexes with antibodies, facilitated intracellular cholesterol accumulation.

Klimov et al. have demonstrated that mouse macrophages cultured in the presence of immune complexes containing LDL and rabbit anti-human LDL antibodies demonstrated an increased uptake of LDL [42]. They have also shown that incubation of human peritoneal macrophages with autologous LDL-containing immune complexes causes transformation of macrophages into foam cells [38].

Griffith et al. [43] have found that human macrophages incubated* in vitro *with insoluble LDL-containing immune complexes accumulate cholesterol and are transformed into foam cells.

It should be mentioned that the LDL of immune complexes amounts to not more than 2% of the total circulating LDL pool [4, 5]. However, this LDL fraction is fundamental to the serum atherogenicity manifested at the cellular level. In a cell culture, immune complexes isolated from the serum cause atherosclerosis-related changes similar to those caused by the whole serum [4, 5]. There is a direct correlation between the LDL content of circulating immune complexes and serum atherogenic potential [4].

Orekhov et al. [44] have shown that insoluble immune complexes containing LDL and heterologous anti-LDL antibodies induce lipid accumulation in cultured cells. The ability of antibodies to stimulate lipid accumulation was found to be dependent on the LDL content of the immune complex [44]. It was also shown that the atherogenic potential of desialylated LDL isolated from the blood of atherosclerotic patients is markedly increased if the LDL forms an immune complex with autoantibodies [2]. The addition of desialylated LDL and anti-LDL autoantibodies to cultured human aortic smooth muscle cells enhanced the intracellular cholesterol accumulation considerably [2, 45, 46]. Native LDL that did not induce intracellular cholesterol accumulation became atherogenic after interaction with autoantibodies (i.e., the LDL acquires the ability to increase the cholesterol content of cultured cells) [2, 45, 46]. Interaction of LDL with anti-LDL autoantibodies considerably increases the uptake (binding and internalization) of the lipoprotein by arterial cells, which may account for the stimulating effect of antibodies on the LDL-induced accumulation of intracellular cholesterol [2]. After being added to a cell culture together with antibodies, fibronectin and Clq complement component (which are the constituents of an immune complex) increase the LDL uptake to a greater extent than the antibodies added alone. This leads to a massive cholesterol accumulation [2].

It was demonstrated that removal of IgG and IgM as well as circulating immune complexes from atherogenic sera of CHD patients leads to a partial or complete elimination of their atherogenic properties (i.e., its ability to induce intracellular lipid accumulation). Removal of immunoglobulin G caused the greatest fall in the serum atherogenicity; the fall was lower after removal of immunoglobulin M, and atherogenicity remained virtually unchanged after immunoglobulin A removal [3]. These facts lead to the suggestion that the majority of the atherogenic LDL of an immune complex is bound with antibodies of the immunoglobulin G class; there is no evidence that IgG-containing LDL-CIC may be more atherogenic and those results only allow assuming that just IgG and, in lesser extent, IgM but not IgA participate in LDL-CIC formation. At the same time, circulating immune complexes (CIC) isolated from these sera brought about accumulation of cholesterol in cultured SMC of unaffected human aortic intima. The ability of atherogenic sera to stimulate the accumulation of intracellular cholesterol correlated with the cholesterol level in the CIC isolated from these sera. The cholesterol content in CIC isolated from sera of CHD patients, which displayed atherogenic properties in culture, was characterized by an elevated cholesterol level in CIC (33.2 ± 1.2 pg/mL) significantly (*P* < 0.01) different from the values seen in the group of healthy donors. Neither of the nonatherogenic sera had an elevated CIC-cholesterol level. These findings suggest that in most cases the cholesterol-containing immune complexes are responsible for the atherogenic properties of the serum [3]. Basing on the knowledge of the absence of atherogenicity of native LDL, it can be suggested that nonmodified LDL does not produce an immune response, and for the formation of LDL-CIC lipoprotein particles should be modified in some way; apo-B desialylation may act as one of the mechanisms of LDL immunogenic modification. At the same time, it is unknown whether LDL-CIC contain only modified LDL, or also native LDL; the last is possible due to common immunoglobulin-binding sites, which may be present in both native and modified LDL.

The mechanisms of intracellular lipid accumulation caused by LDL-CIC obviously are an area that needs further investigation. There is evidence that immune complexes formed between modified LDL as antigens and IgG autoantibodies may modulate the inflammation in atherosclerosis via Fc receptor signaling and complement activation; the role of antibody isotypes in atherogenesis is unclear, since IgG is regarded as potentially proatherogenic, and IgM may even play a protective role [47]. The last finding contradicts with earlier data on atherogenicity of IgM-containing LDL-CIC; however, till now there is no evidence that activation of inflammation and foam cells formation should go in parallel. It is not definitely known in which way foam cells formed as a result of intracellular lipid accumulation further drive the progression of atherosclerosis; there exists the possibility of macrophages to acquire proinflammatory phenotype after ingesting LDL-IgG through Fc gamma receptor. However, it is known that LDL-CIC may induce other atherosclerosis-related processes at the cellular level, namely, excessive production of connective tissue matrix and cellular proliferation [5, 36]. It has been shown recently that* in vitro* produced immune complexes containing oxidized LDL stimulate type IV collagen production by mesangial cells, the effect being realized via Fc gamma receptors I and III [48]. Such immune complexes also increased proliferative activity of cultured human monocytes, and this effect was mediated by cross-linking of Fc gamma receptor I; a concentration-dependent production of monocyte colony-stimulating factor was observed. These results offered a novel mechanism by which an immune reaction toward modified LDL can play a role in local accumulation of macrophages in atherosclerotic lesions [49]. It is generally approved that LDL-CIC effects are mediated via interaction of immunoglobulin moiety with Fc receptors; since IgG and IgM antibodies do not share the same receptors, this may be the explanation for the abovementioned difference in uptake of LDL bound either to IgG or to IgM.

It has been demonstrated that the lipoprotein-antibody complexes prepared* in vitro* affect lipoprotein metabolism in human fibroblasts and monocytes [50, 51] and facilitate the accumulation of lipids in mouse macrophages [52]. Complexes of human LDL with polyclonal goat antibodies against LDL induce the deposition of lipids in cultured SMC of human aortic intima and peritoneal mouse macrophages [3].

Taken together, these findings suggest that multiple-modified desialylated LDL has immunogenic properties and circulating immune complexes containing modified LDL and anti-LDL autoantibodies are the blood components responsible for primary cholesterol accumulation in vascular cells. Since cholesterol accumulation is accompanied by stimulation of other atherosclerotic manifestations at the cellular level, it can be suggested that the presence of LDL-containing complexes in the blood promotes the emergence and development of atherosclerotic lesions in the vessel wall.

## 4. Diagnostic and Prognostic Value of LDL-CIC in Atherosclerosis

Using a simple method of measurement of LDL-CIC level, Orekhov et al. demonstrated that only LDL-CIC level and the apo B/apo A-1 ratio contributed strongly to the discrimination between patients with coronary and/or extracoronary atherosclerosis and those without stenosis [53]. In the same study, total cholesterol, triglycerides, HDL cholesterol, apo B, Lp[a], and apo A1 did not correlate with the presence and severity of coronary and/or extracoronary atherosclerosis. The authors concluded that LDL-CIC level might be the most reliable marker of atherosclerosis as compared to other parameters of lipid profile. LDL-CIC level was significantly correlated also with the severity of coronary atherosclerosis, and this biochemical parameter was proposed to be used as a sensitive and specific marker for atherosclerosis, possessing a high diagnostic value [5, 53, 54]. Salonen et al. have reported that the titer of antibodies against MDA-modified LDL in blood serum is associated with the progression of a carotid atherosclerosis [55]. In the recent Epidemiology of Diabetes Interventions and Complications (EDIC) Trial it has been demonstrated that cholesterol and apolipoprotein B content of immune complexes were significantly higher in patients who showed progression of the internal carotid IMT than in those showing no progression, regression, or mild progression, and cholesterol content of immune complexes was a significant positive predictor of internal carotid IMT progression [56]. High cholesterol levels in CIC are considered to be surrogate markers of modified LDL associated with increased carotid intima-media thickness and cardiovascular events. Lopes-Virella et al. have measured oxidized LDL, advanced glycation end products-modified LDL, and malondialdehyde-modified LDL in CIC, determined their relationship with increased carotid IMT in type I diabetes, and compared the strength of the association with that observed with conventional risk factors [57].

The most recent evaluation of diagnostic and predictive role of LDL-CIC (immune cholesterol) as well as other lipid parameters in early carotid atherosclerosis was carried out by Sobenin at al. in two-year prospective study [58]. The rate of atherosclerosis progression was estimated by high-resolution B-mode ultrasonography as the increase in intima-media thickness (IMT) of common carotid arteries. The patients with elevated levels of LDL-CIC were characterized by significantly higher levels of serum total and LDL cholesterol as well as significantly increased mean and maximum intima-media thickness of common carotid arteries. Cholesterol level of LDL-CIC and serum LDL cholesterol were contingent with the extent of early carotid atherosclerosis (*P* = 0.042 and *P* = 0.049, resp.). Additionally, LDL-CIC was characterized by the highest values of sensitivity and specificity as compared to commonly used lipid parameters. Only LDL-CIC, but not any other lipid parameter, was contingent with the progression of early carotid atherosclerosis (*P* = 0.042) and also had the highest levels of relative risk and odds ratio [59]. Normal level of LDL-CIC (below 16.0 *μ*g/mL) was the only parameter that predicted the absence of carotid atherosclerosis progression for two following years at prognostic value of 78.3% (95% CI, 67.1–87.3) [59]. Normal levels of serum total cholesterol, LDL and HDL cholesterol, and triglycerides did not possess statistically significant predictive values. Thus, in spite of the absence of clinical manifestations of atherosclerosis, the elevated level of LDL-CIC is associated with increased intima-media thickness and can be regarded as a predictor for higher risk of atherosclerosis development [58, 59].

In large meta-analyses of prospective population studies in 165,544 participants without baseline CVD in 37 prospective cohorts (calendar years of recruitment: 1968–2007) with up to 15,126 incident fatal or nonfatal CVD outcomes (10,132 CHD and 4994 stroke outcomes) during a median follow-up of 10.4 years (interquartile range: 7.6–14 years), both baseline LDL and HDL cholesterol levels were strong predictors of both CHD and stroke [60]. The studies included in this cited meta-analysis described conventional lipid profile parameters, but not LDL-CIC, as predicting variables. However, the results of analysis demonstrate that the predictive biomarkers in healthy population are not the same as those for the diagnosis and prognosis of atherosclerosis; therefore, diagnostic and prognostic role of LDL-CIC should be in focus for further investigations in both nonatherosclerotic subjects and preclinical and overt atherosclerosis.

## 5. Conclusions

**Figure 1:**
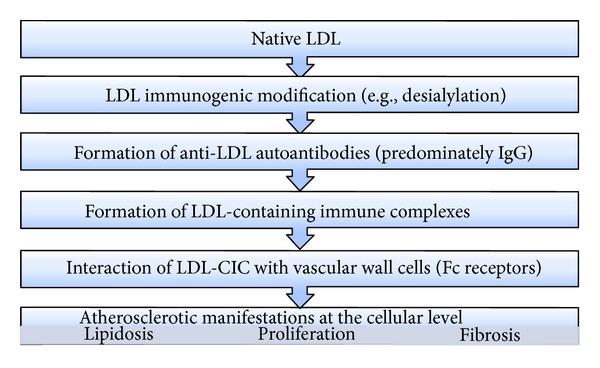
Schematic presentation of the role of lipoprotein-containing immune complexes in atherogenesis.

On the basis of current data, it is possible to define the role of lipoprotein-containing immune complexes in atherogenesis (Figure 1). It can be supposed that LDL-CIC may play a significant role in atherogenesis and are involved in the formation of early atherosclerotic lesion. LDL-CIC can induce massive cholesterol accumulation in cultured vascular cells that leads to foam cell formation, cellular proliferation, and extracellular matrix production [5, 36]. Modified LDL (e.g., desialylated, glycosylated, and oxidized LDL), which has an atherogenic potential in contrast to native LDL, appears in the blood. It stimulates atherosclerotic manifestations at the arterial cell level, for example, by inducing the intracellular lipid accumulation. Atherogenic modified LDL triggers the production of anti-LDL autoantibodies which react with LDL, leading to the formation of an LDL-containing immune complex. Interaction of anti-LDL antibodies with modified LDL increases their atherogenic potential. After forming an immune complex with anti-LDL antibodies, the originally nonatherogenic, native LDL becomes atherogenic (i.e., they are capable of inducing intracellular lipid accumulation and other atherosclerosis-related alterations). On entering the subendothelial space of the arterial intima and interacting with subendothelial cells, lipoprotein-containing immune complexes may induce the whole spectrum of atherosclerotic cellular perturbations. Titer of LDL-CIC in blood serum significantly correlates with progression of IMT and has the highest diagnostic value among other measured serum lipid parameters. After removal of CIC, the sera lose their atherogenic properties. Elevation of CIC-cholesterol seems to be a characteristic feature of coronary atherosclerosis while CIC-cholesterol might well be a possible risk factor.

## Figures and Tables

**Table 1:** The properties of LDL from circulating immune complexes.

Characteristic	LDL from CIC compared to native circulating LDL
Neutral lipid content (free cholesterol, esterified cholesterol, and triglycerides)	Lowered
Phospholipid content	Lowered
Sialic acid content	Lowered
Neutral sugars content	Lowered
Electrophoretic mobility	Increased
Hydrated density	Increased
Particle size	Decreased

## Abbreviations

CHD: Coronary heart disease
CIC: Circulating immune complexes
HDL: High density lipoprotein
LDL: Low density lipoprotein
LDL-CIC: LDL-containing circulating immune complexes
MDA: Malondialdehyde
SMC: Smooth muscle cells
IMT: Intima-media thickness.
